# Urobiome: In Sickness and in Health

**DOI:** 10.3390/microorganisms7110548

**Published:** 2019-11-10

**Authors:** Bartosz Wojciuk, Agata Salabura, Bartłomiej Grygorcewicz, Karolina Kędzierska, Kazimierz Ciechanowski, Barbara Dołęgowska

**Affiliations:** 1 Department of Immunological Diagnostics, Pomeranian Medical University in Szczecin, 70-123 Szczecin, Poland; 2 Clinic of Nephrology, Internal Medicine and Transplantation, Pomeranian Medical University in Szczecin, 70-123 Szczecin, Poland; silverata@gmail.com (A.S.); karolina.kedzierska@gmail.com (K.K.); kazcie@pum.edu.pl (K.C.); 3 Department of Laboratory Medicine, Pomeranian Medical University in Szczecin, 70-123 Szczecin, Poland; b.grygorcewicz@gmail.com (B.G.); barbara.dolegowska@pum.edu.pl (B.D.)

**Keywords:** urobiome, virome, chronic kidney disease

## Abstract

The human microbiome has been proven to contribute to the human condition, both in health and in disease. The metagenomic approach based on next-generation sequencing has challenged the dogma of urine sterility. The human urobiome consists of bacteria and eukaryotic viruses as well as bacteriophages, which potentially represent the key factor. There have been several significant findings with respect to the urobiome in the context of urological disorders. Still, the research on the urobiome in chronic kidney disease and kidney transplantation remains underrepresented, as does research on the role of the virome in the urinary microbiota. In this review, we present recent findings on the urobiome with a particular emphasis on chronic kidney disease and post-kidney transplantation status. Challenges and opportunities arising from the research on the human urobiome will also be discussed.

## 1. Introduction

The microbiome represents the overall set of microorganisms existing in the human body. Since the discovery of bacteria, it has been known that the human organism coexists with microbial ecosystems in body sites like skin, mucous membrane, and particularly the gastrointestinal tract. There is also consensus that these microorganisms contribute significantly to the development of immunity and other physiological functions (e.g., clotting via vitamin K production). The immunological function of what was previously referred to as “normal flora” was mostly attributed to the competition between native and foreign agents. Therefore, the postulate of reasonable antibiotic use has emerged in order to protect this potential. This was emphasized by the clinical evidence for the consequences of native microbial flora imbalance, as clearly revealed in colitis related to *C. difficile*.

Currently, it is estimated that ten bacterial cells associate with each human cell, such that the human body appears to be one huge ecosystem containing a dynamic balance within it [1]. Furthermore, it has been hypothesized that the beneficial functions of the microbiota are not only the result of competition between microbial species or the side-products of their metabolism, but are also caused by direct interactions between microorganisms and host cells. Hence, these interactions modulate the functions of the host cells. This assumption has provided the starting point for extensive research on the microbiota. Nevertheless, this would have not been possible had it not been for the development of new analytical methods.

Next-generation sequencing (NGS), invented nearly a decade ago, transferred the genetic approach to the genomic level. This was achieved by targeting whole-genome regions or simply entire genomes. In terms of microbial research, it has been assumed that the sequencing of highly variable fragments encoding the 16S subunit of ribosomal RNA (V1–V9 regions) enables the identification of bacterial species without the necessity of culturing. This has been a milestone for metagenomics (i.e., a branch of NGS-based research) [2,3]. Consequently, the term *microbiome* has emerged, and this should be understood as referring to the whole set of microbial DNA identified on different phylogenic levels, mirroring the composition of the microbiota in particular niches.

Using this methodology, the Human Microbiome Project (HMP) was initiated [4]. The HMP was limited by the existing dogma that particular body sites remained sterile; therefore, the HMP targeted the gastrointestinal tract, the oral cavity, and skin, but not urine. Nevertheless, our understanding of the urinary tract milieu/microbiome developed simultaneously. Urinary tract infections (UTIs) have always been a significant clinical issue. Additionally, due to the fact that UTIs represent the most common clinical form of nosocomial infection, UTI epidemiology mirrors the drug resistance status of certain hospital environments. Routine diagnostic procedures are based on urine sample culture with the use of blood agar as well as MacConkey agar. Positive samples above the 10^5^ colony-forming units (CFU)/mL threshold level prove the efficiency of the diagnostics for evident clinical cases caused by fast-growing, strictly pathogenic strains like uropathogenic *Escherichia coli.* Still, a spectrum of clinical syndromes like urinary discomfort, urinary urgency, or pelvic pain that do not meet the infection criteria as described above continue to pose challenges for physicians. Microbiologically, these results can be interpreted either as negative cultures or as contamination. It was then hypothesized that the uncertain status of such patients could be a result of the insufficiency of the routine microbiological methods used. Hence, it was proposed to increase the urine volume, enlarge the number of culture media used, and extend the culturing time in order to enhance the culturing of slow-growing bacteria. These methodologies were referred to as enhanced urine culture techniques (EUCTs), and achieved promising outcomes. However, they have never been implemented in practice [1,5]. The urine sterility dogma fell when NGS-based metagenomic studies revealed the presence of microbial communities not only in pathologies but also in asymptomatic individuals. Furthermore, it is now clear that the urinary microbiome—the urobiome—contains not only bacteria, but also a variety of viruses. What makes this issue more complex is that the viruses that contribute to the urobiome represent either eukaryotic viruses or bacteriophages (or phages, for short), thus representing two types of lifecycle—lytic and lysogenic [6]. As a consequence, the interplay between bacteria and their viruses (phages) is attracting growing attention from researchers.

Not surprisingly, the significance of the microbiome in different pathologies has quickly become an emergent target for research activity. It was first confirmed in terms of gastrointestinal tract and autoimmune diseases, with Crohn’s disease as a major example. Furthermore, it has been found in other seemingly distant pathologies, such as mental disorders [7,8].

Analogously, the urobiome started being subjected to investigation by urologists in relation to the pelvic complaints listed previously, and these will be further discussed below. Nevertheless, there are other complex conditions in which the urobiome deserves attention. These include chronic kidney disease, in particular with regard to post-kidney transplantation status.

In this review, we will focus on recent advances in urobiome research, with an emphasis on nephrological aspects like chronic kidney disease and kidney transplantation. Additionally, we will address the significance of the viral components of the human urobiome.

## 2. Bacterial Components of Urobiome

### 2.1. Urinary Microbiome in Health

There are several issues that need to be addressed before discussing the role of the urinary microbiome in the specified conditions. Metagenomic analyses deliver large volumes of data and require further analysis. Apart from microorganism phylum, genus, or species identification, these also provide quantitative information about the diversity of organisms and the contribution of particular bacteria to the microbiome. Diversity is normally interpreted within a single sample, recognized as alpha diversity, or between samples (i.e., beta diversity). Alpha diversity is measured with a set of indexes, while beta diversity is measured using dis/similarity distance, such as Bray–Curtis dissimilarities, Jaccard distance, or UniFrac distance, and then visualized using NMDS (non-metric multidimensional scaling) or commonly PCoA (principle coordinate analysis). As a consequence, at least three parameters are used to characterize the urobiome: composition and dominant microorganisms (if possible, to recognize), diversity, and clusters related to certain conditions. 

It is also necessary to emphasize that urine collection significantly influences the understanding of the urobiome as a whole. Studies on urobiome in asymptomatic individuals target voided urine that contains microorganisms from the lower urinary and genital tract. Therefore, it is postulated to consider this not only as the urobiome but rather the genitourobiome—especially in women [9]. On the other hand, there are characteristic clinical conditions such as bladder cancer that require targeting the pure bladder urine collected via suprapubic aspiration. This is applied in the studies on bladder pathologies but creates ethical questions in investigating asymptomatic individuals. The bladder urobiome and possible differences between the urobiome and genitourobiome will be discussed in further sections.

As the research on the urinary microbiome is still developing, it is challenging to recognize a “properly composed urobiome”. This problem has been addressed in terms of the intestinal microbiome, which has revealed the presence of so-called *enterotypes*—a set of microbiome clusters different in composition, dominant species, and diversity [10]. The amount of data on healthy individuals’ urobiomes is highly limited, but this approach has also been implemented. Remarkably, most of the studies focus on gender differences in the urotypes. Due to the limited number of cases involved in each study, a further distinction based on other variables like race, geographical location, etc. currently appears challenging. The study of Gottschick et al. investigated women with bacterial vaginosis and indicated eight different urotypes (UTs) in women and urobiome characteristics for men. In this study, asymptomatic women presented higher urobiome diversity compared to those suffering from bacterial vaginosis. All urotypes were clustered according to the abundance of particular species, respectively: *Prevotella amnii, Sneathia amnii, Gardnerella vaginalis,* and *Atopobium vaginae* (UT1), *Lactobacillus iners* (UT2), *Enterobacteriaceae* (UT3), *Enterococcus faecalis* (UT4), *Streptococcus agalactiae* (UT5), *Citrobacter murliniae* (UT6), and *Lactobacillus crispatus* (UT7). No dominant organism was identified in UT8 [11]. All urotypes except for UT7 were indicated in both symptomatic and asymptomatic women, while U7 was only found in healthy individuals. Respectively, two of the urotypes—UT2 and UT7—were present exclusively in women. Consistently, Fouts et al. indicated two healthy-state-associated urotypes: one in women, dominated by *Lactobacillus,* and one in men, dominated by Gram-positive bacteria [12]. Other studies, cited by Mueller et al. [10], also identified separate female urotypes dominated by *Lactobacillus, Gardnerella, Corynebacterium, Streptococcus,* and *Staphylococcus*. In contrast, Siddigni et al. did not recognize any characteristic urotypes; however, consistent with other authors, they indicated the predominance of *Lactobacillus, Gardnerella,* and *Prevotella* in female urobiomes [13]. Lewis et al. [14] investigated urinary microbiota in asymptomatic individuals with pyrosequencing (i.e., less-advanced technology than NGS). These findings were consistent with the others in terms of the increased diversity of the female urobiome in comparison to males. The correlation of urobiome diversity with age appeared interesting. Diversity decreased but new genera like *Jonquetella, Parvimonas, Proteiniphilum,* and *Saccharofermentans* were detected in the people aged above 70 years. The aforementioned findings are systematically displayed in Table 1.

### 2.2. Urinary Microbiome in Disease

As mentioned previously, microbiome issues have mostly been addressed from the urological point of view, so the significant set of data refers to pathologies such as bladder cancer, pelvic pain syndromes, urinary incontinence, prostatic biopsy, and sexually transmitted infections (STIs) [15]. Complex questions about the urinary tract infections and antibiotic treatment seem to be strongly related to urobiome dysbiosis. Mulder et al. indicated that antibiotic treatment influences the urobiome in older adults, regarding its composition but not its alpha diversity. The study found that antibiotic treatment decreased the total contribution of *Lactobacillus* and *Finegoldia* in favor of *Escherichia coli* [16]. However, the causative connection between urobiome imbalance and infection remains unclear. Precisely, the question of whether the imbalance exists prior to infection or represents a side-effect of antibiotic treatment has not been fully resolved. Nevertheless, the proven imbalance of pre-existing infection would possibly put a new light on the whole understanding of the cut-off between health and pathology. This viewpoint is consistent with the study of Nelson et al., where the differences in urobiome composition in terms of sexually transmitted diseases were indicated. It was shown that men with STI-positive anamneses represented urobiomes abundant in anaerobic bacteria, although diversity among all samples remained significant [17]. It should become considerable to recognize a sort of continuum between health, asymptomatic imbalance, and symptomatic urinary tract infection. The aforementioned study of Gottschick et al. [11] also indicated differences between the urobiomes of healthy individuals and women treated with metronidazole for bacterial vaginosis. In contrast, in this study the differences referred mostly to diversity, which was the highest in health, decreased in bacterial vaginosis, and was highly reduced during metronidazole treatment [10]. Bladder pathologies remain an interesting context to investigate the urobiome. In the case of bladder cancer, it has been proven that the bladder microbiome differs between patients suffering from bladder tumor and healthy individuals. Additionally, *Fusobacterium* spp. was found to be potentially tumorigenic. At the same time, *Herbaspirillum*, *Porphyrobacter,* and *Bacteroides* represented prognostic potential in terms of recurrence and progression [18,19]. Benign pathologies that significantly affect life quality have also been considered in relation to the urobiome. It has been shown that the urobiome differs between women suffering from urinary incontinence and healthy individuals. Still, it remains a matter of speculation as to what the exact mechanism could be. The bladder–brain axis, analogous to the gut–brain axis, is being proposed [20,21].

### 2.3. Chronic Kidney Disease and Kidney Transplantation

#### 2.3.1. Chronic Kidney Disease

Apart from the urological connotations of previously mentioned studies, there is also a nephrological viewpoint that should be considered. Regarding the complex character of chronic kidney disease, it is highly challenging to investigate connections between kidney pathologies and the urobiome, because the evidence is even more limited in this area. Nevertheless, the study of Kramer et al. indicated differences in urobiome diversity that were dependent on glomerulal filtration rate (GFR) in the course of chronic kidney disease (CKD). The authors focused on individuals with CKD in stages 3 to 5. The cause of CKD was not considered as variable, except for diabetic kidney disease. Eleven different urotypes were identified in all groups—three of these with no domination. What appears significant is that the diversity of urobiomes was positively correlated with eGFR values—the most developed in stage 3, and highly decreased in stage 5 [22].

#### 2.3.2. Kidney Transplantation

The role of urobiome in post kidney transplantation status has not been extensively investigated, although some significant findings should be highlighted. Rani et al. proved that the urobiomes of kidney recipients and healthy individuals differed in terms of their diversity and composition. Urinary microbiota from kidney recipients were less diverse and dominated by potentially pathogenic Gram-negative bacteria like *Escherichia* or *Enterobacter*, whereas healthy controls presented a higher microbiome diversity and higher prevalence of non-pathogenic Gram-positive organisms like *Propionibacterium, Corynebacterium,* and *Mobiluncus.* These differences appeared independent of kidney disease preceding transplantation [23]. Fricke et al. [24] prospectively analyzed kidney recipients. They discovered that urobiomes appeared highly diverse in the recipients at the moment of transplantation, became similar one month post transplant, and this similarity persisted until six months post transplant [24]. The composition of urobiomes appeared independent of kidney allograft function, apart from Bifidobacteriales, which was associated with increased creatinine levels [24]. Despite their significance, these findings do not resolve the question of the clinical significance of urobiome variability after kidney transplantation. The metagenomic approach to this issue will be discussed below. Still other studies addressed the problem at the clinical level. Wu et al. [25] investigated the urobiomes in recipients with decreased allograft function, which was recognized as a 25% increase in creatinine level in relation to 3 months post-transplant baseline. *Corynebacterium* spp. was more prevalent in individuals with chronic allograft dysfunction [25]. However, this study was not prospective and was focused on the creatinine level as an indicator of allograft dysfunction with no consideration of histopathological lesions in the allograft. The correlation between interstitial fibrosis/tubular atrophy (IFTA) in allograft biopsies and urobiome composition was considered in the study by Modena et al. It was stated that *Lactobacillus* spp. dominated in healthy females and female recipients pre transplant, respectively *Streptococcus* spp. in analogy to male groups. The contributions of both genera appeared negatively correlated with the IFTA intensity assessed in the kidney allograft biopsies and this was more apparent in males [26]. The findings described above are systematically displayed in Table 2. 

The aforementioned dominance of pathogenic Gram-negative bacilli in post-kidney transplant urobiomes [23] points to the issue of urinary tract infections in the recipients. These are most frequently caused by *E. coli.* The opinions on the role of UTI in shaping the long-term allograft function are not consistent. However, more recent studies strongly suggest the negative influence of either early or late UTIs on kidney allograft function [27,28,29].

None of the cited studies makes distinctions with regard to either the immunosuppressive regimen or to the underlying chronic kidney disease pre transplant. Immunosuppression implemented in the recipients enrolled in these analyses was based on calcineurin inhibitors, mycophenolate, and steroid-based protocols.

## 3. Viruses of the Urinary Tract

### 3.1. Urinary Virome in Health

The amount of data relevant to the human urinary virome is highly limited. Nevertheless, it has been confirmed that the kidneys and lower urinary tract are colonized by different viruses [30]. The large cohort analysis performed on 142 kidney recipients and the same number of healthy volunteers revealed 37 unique viruses, 29 of which were first identified in human urine samples [31]. Perfectly adapted viruses are clinically silent, which makes them unexpected. Pathogenic eukaryotic viruses that cause symptoms like diarrhea or cough increase their chance of transmission. On the other hand, these new orphan viruses discovered by new sequencing techniques show that keeping silent enables them to establish an ideal equilibrium between the virus and host, with potential advantages for both [32]. The JC virus (JCV) represents an evident example. It has been estimated that in the African American population, approximately 30% of individuals express active JCV replication in the urine. Researchers point out that replication of this virus is associated with lower rates of nephropathy [30]. Other studies target exclusively eukaryotic viruses: human papillomaviruses (HPVs), BK virus (BKV), JC virus, and Torque Teno virus (TTV). Apart from the definite clinical significance of these, such an approach does not consider the context of the whole virome, which also containes the bacteriophages as a huge component [33,34].

### 3.2. Urinary Virome in Diseases

The human virome is connected with many different diseases, such as periodontal diseases, HIV infection, cystic fibrosis, inflammatory bowel disease, and urinary tract infections. There are viruses involved in carcinogenesis, both as temporary and permanent elements of the human virome. The relationship between carcinogenesis and urinary virome is not yet fully understood. Studies on the intestinal virome indicate that the virome influences cancer development [35]. The majority of recognized viruses remain in latent state and reactivate under immunosuppression, which results either from HIV infection or organ transplantation. Therefore, viruses are constantly gaining the interest of transplant researchers.

#### Urinary Virome and Kidney Transplantation

It is well known that viruses after kidney transplantation are one of the major causes of graft loss after kidney transplantation [36,37,38]. BK polyomavirus nephropathy (BKVN) represents a great challenge for kidney recipients. An aforementioned member of the polyomavirus family, JCV, is associated with progressive multifocal leukoencephalopathy. However, JCV viruria has been considered as a protective factor against acute rejection and allograft failure [39]. In a study performed on 22 patients, Rani et al. revealed that all recipients expressed BKV replication in the urine, independent of their viremia status. Other polyomaviruses (i.e., JCV and TTV) were also present. The viruses were polymorphic. Polymorphisms were mostly connected with VP1 and VP2 proteins and large T antigens, indicating the varied pathogenicity of the viruses analyzed. Although BKV dominated in the virome of BKV-positive individuals, viral families other than *Polyomaviridae* were also detected: *Anelloviridae, Adenoviridae, Herpesviridae*, *Papillomaviridae,* and some unclassified ones. Significantly, lower total counts of BKV were found in the group classified as serum BKV PCR- negative. In the case of JCV and TTV, the differences between those groups were not statistically significant. *Anelloviridae* were more prevalent in the samples with fewer polyomaviruses (BKV, JCV, TTV). Common viruses such as Epstein–Barr virus, cytomegalovirus, and herpes simplex viruses (HSVs) were not detected in this study [40]. Another study of Schreiber et al. used metagenomic virome sequencing and found JCV in the urine of kidney donors and recipients at the time of transplantation as well as in recipients 4–6 weeks and/or 1 year post transplant. However, despite JCV positivity at the time of transplantation, phylogenetic analysis revealed the domination of donor-derived JCV strains [41]. The study of Santiago-Rodriguez et al. [34] on patients with urinary tract infection (UTI) did not establish a significant difference between the virome from UTI patients and those without UTIs. Both groups were abundant in HSV: polyomaviruses and HPV. There were no gender-specific differences in the viral community between individuals studied [34].

### 3.3. Bacteriophages in the Urinary Tract

Bacteriophages play an important role in the dynamics of the bacterial community, and their abundance within some niches of the human body is well studied. The literature suggests that in the gut microbiome, bacteriophages might contribute to the stabilization of the beneficial bacterial community and may also provide the innate defense against pathogenic bacteria [42,43,44,45,46,47]. Brown-Jaque et al. [48] showed that bacteriophages infecting *E. coli* were present in 46.1% (16) of analyzed urine samples when analyzed with the use of the plating technique. Note that the potential application of bacteriophage for the treatment of urinary tract infection has also been described [49,50]. Unfortunately, the role of bacteriophages in the urobiome is still unknown [51]. It is worth emphasizing that bacteriophages appear to be the most abundant members of the human virome [52]. Santiago-Rodriguez suggests that bacteriophages are characterized by a primary lysogenic lifecycle in the urinary virome. This suggestion was supported by a high proportion of identified integrases compared to other phage genes [34]. Their results showed that 27% of the contigs were homologous to known viruses; in fact, 99% of those sequences representing bacteriophages.

## 4. Fungi in the Urobiome

Very little is known about components of urobiome other than the bacteriome. So far, no published study has characterized the urinary mycobiome with the use of next-generation sequencing technology [53]. Mycobiome analysis is limited by current technologies. Especially, the non-fungal host DNA may interact with the reagent used for mycobiome DNA isolation and amplification. Reports are focused on the optimization of fungal DNA extraction methods from urinary samples for their future processing [54]. Some reports showed the viability of *Candida* spp. isolated from urine samples with the use of standard culture methods [5,55,56,57]. Another study showed the detection of fungi in patients with urologic pelvic pain syndrome, in which a biosensor system was used [58]. This report showed an increase in the percentage of patients in which fungi were detected: from 3.9% (asymptomatic) to 15.7% (symptomatic). The urinary mycobiome, as an important part of the human microbiota, is ripe ground for future studies.

## 5. Challenges and Future Directions

The metagenomic approach has become a milestone in investigating the human microbiota as it enables culture-independent approaches. It has paved the way for cutting-edge ideas in milieus that have traditionally been considered sterile, such as urine. Still, there are some limitations to the most popular metagenomic approach, which targets 16S coding regions. The most significant limitation is that one retrieves data on phylum-, genus-, or species-related sequences, with no verification of the status of the whole organisms (i.e., whether they are alive or dead). Secondly, the outcomes are critically influenced by sample collection procedure (i.e., the already mentioned suprapubic aspiration vs. voided urine collection) as well as the applied DNA isolation protocol and bioinformatics platforms [59]. As a result, the outcomes from different research centers are hardly comparable. This is even more apparent in terms of the virome. Finally, there is still a gap in understanding the exact model by which the urobiome influences the host and, therefore, clinical conditions. Regarding the vital status of particular microorganisms recognized in the urobiome, it is suggested that NGS and EUCTs are complementary [9]. In this manner, EUCTs may represent a tool to verify the status of the microbiota. Additionally, the cultures obtained via EUCT provide further data such as whole-genome profiles that have already been used to specify the differences and overlap between urinary, vaginal, and enteric microbiota [60]. Potentially, these can be also applied for an in-depth analysis of the functional status of the microbiome. This can also be targeted by NGS, but this must implement the shotgun approach rather than 16S sequencing. In this case, a larger spectrum of genes can be identified, and their ontology can be verified in gene libraries like the Kyoto Encyclopedia of Genes and Genomes (KEGG). This idea has been implemented in the aforementioned study of Rani et al., in which the shotgun approach revealed the presence of genes related to sulfamethoxazole/trimethoprim (SXT/TMT) resistance in the urobiomes from kidney recipients routinely treated with STX/TMT prophylaxis [23]. Despite some significant findings on the urobiome structure which have been obtained in recent years, it is still challenging to draw general conclusions. It appears to be quite evident that *Lactobacillus* spp. significantly contribute to the urobiome of healthy women and that the urobiome changes in the course of bladder cancer, possibly with some prognostic implications. Still, the whole groups of eukaryotic components of the urobiome (e.g., fungi and protozoa) have not yet been targeted. This is mostly due to challenges in DNA extraction and further analysis. The urobiome in chronic kidney disease and after kidney transplantation still needs more extensive and prospective research to create general conclusions. This is highly relevant with regard to the differences in immunosuppression and other variables which have not yet been considered. Special attention should be paid to the urinary virome post transplant. In the authors’ opinion, it seems reasonable to implement a complex multi-omics approach, which would include metagenomics, possibly proteomics, and metabolomics, in order to identify the network between bacterial and viral components of the urobiome and their interplay with accompanying urinary tract tissues.

## Figures and Tables

**Table 1:** Urobiome differentiation in healthy individuals according to gender and age. ^1^ Based on 16S ribosomal subunit gene next generation sequencing; ^2^ based on pyrosequencing.

Gender	Overall ^1^	Age 20–49 ^2^	Age 50–69 ^2^	Age > 70 ^2^
Female	*Lactobacillus, Gardnerella, Prevotella, Sneathia, Atopobium, Enterococcus, Streptococcus, Enterobacteriaceae*	***Azospira*** ***Butyricicoccus*** ***Coriobacterium*** ***Friedmanniella*** *Gardnerella* ***Microvirgula*** *Neisseria* *Paraprevotella* ***Rhodopila*** ***Sutterella*** ***Tepidimonas*** ***Tessaracoccus***	*Brevibacterium* ***Catonella*** ***Caulobacter*** ***Methylovirgula*** ***Pelomonas*** *Peptostreptococcus* ***Sneathia*** ***Streptophyta*** ***Thermoleophilum***	*Actinomyces* *Arthrobacter* ***Gulosibacter*** ***Jonquetella*** ***Lachnospiracea incertae sedis*** ***Modestobacter*** ***Oligella*** ***Parvimonas*** ***Proteiniphilum*** *Rhodococcus* ***Saccharofermentans***
Male	*Lactobacillus, Sneathia, Veillonella, Corynebacterium Prevotella, Streptococcus, Ureaplasma*	*Pseudomonas, Lactobacillus, Actinobaculum,*		*Aminobacterium* *Anaerococcus* *Anaerophaga* *Anaerosphaera* *Anaerotruncus* *Atopobium* *Atopostipes* *Azospira* *Butyricicoccus* *Campylobacter* *Catonella* *Corynebacterium* *Dialister* *Eubacterium* *Filifactor* *Finegoldia* *Fusobacterium* *Lactonifactor* *Marixanthomonas* *Megasphaera* *Microvirgula* *Mobiluncus* *Murdochiella* *Mycoplasma* *Parvimonas* *Peptococcus* *Peptoniphilus* *Peptostreptococcus* *Porphyromonas* *Prevotella* *Proteiniphilum* *Pseudoramibacter* *Rikenella* *Saccharofermentans* *Sediminitomix* *Lactobacillus* *Actinobaculum*

**Table 2:** Urobiomes in different stages of chronic kidney disease (CKD). IFTA: interstitial fibrosis/tubular atrophy.

Clinical Status	Microorganisms	Methods
CKD stage 3–5	*Staphylococcus, Lactobacillus, Enterobacteriaceae, Gardnerella, Prevotella, Streptococcus, Corynebacterium, Aerococcus, Anaerococcus, Bifidobacterium,*	V4 region 16s RNA gene sequencing
Kidney recipients before transplantation	*Anaeroglobus, Achromobacter, Clostridiaceae, Dethiosulfovibrio, Oligella, Massilia, Microvirga, Pseudoramibacter, Sneathia, Staphylococcus*	V1–V3 region 16s RNA gene sequencing
Males 1 month post transplant—stable function vs. healthy controls	*Gardnerella, Prevotella, Corynebacterium, Lactobacillus, Streptococcus (reduced)*	V2,3,4,6,7,8,9 region 16s RNA gene sequencing
Males 1 month post transplant—IFTA vs. stable function	*Prevotella, Corynebacterium, Lactobacillus, Streptococcus*	V2,3,4,6,7,8,9 region 16s RNA gene sequencing
Females 1 month post transplant—stable function vs. healthy controls	*Gardnerella, Prevotella, Corynebacterium (reduced), Lactobacillus*	V2,3,4,6,7,8,9 region 16s RNA gene sequencing
Females 1 month post transplant—IFTA vs. stable function	*Gardnerella, Prevotella, Lactobacillus*	V2,3,4,6,7,8,9 region 16s RNA gene sequencing
Males 6 months post transplant—stable function vs. healthy controls	*Prevotella, Corynebacterium, Lactobacillus, Streptococcus*	V2,3,4,6,7,8,9 region 16s RNA gene sequencing
Males 6 months post transplant—IFTA vs. stable function	*Gardnerella, Prevotella, Corynebacterium, Lactobacillus, Streptococcus (reduced)*	V2,3,4,6,7,8,9 region 16s RNA gene sequencing
Females 6 months post transplant—stable function vs. healthy controls	*Gardnerella, Prevotella, Lactobacillus*	V2,3,4,6,7,8,9 region 16s RNA gene sequencing
Females 6 months post transplant—IFTA vs. stable function	*Gardnerella, Prevotella, Lactobacillus*	V2,3,4,6,7,8,9 region 16s RNA gene sequencing
Kidney recipients first 12 months post transplant vs. healthy controls	*Streptococcus, Enterococcus, Escherichia, Propionibacterium, Ralstonia, Proteus, Bacteroides, Salmonella, Shigella, Lactobacillus*	Shotgun sequencing
Kidney recipients with decreased graft function at least 12 months post transplant vs. controls with stable graft function	*Corynebacterium, Rhodococcus, Parabacteroides, Staphylococcus, Planococcaceae, Facklamia,*	V4, ITS1, ITS2 regions 16s RNA gene fragment sequencing
